# Supplementary material for: Effects of a Chair Positioning Session on Awake Non-Intubated COVID-19 Pneumonia Patients: A Multicenter, Observational, and Pilot Study Using Lung Ultrasound
Source: J Clin Med. 2022 Oct 5;11(19):5885. doi: 10.3390/jcm11195885 (PMC9573720; doi:10.3390/jcm11195885)
Supplement: Supplementary file 1 [file jcm-11-05885-s001.zip › jcm-1913116-supplementary.pdf]

Journal of
Clinical Medicine

Supplementary Figures and Tables

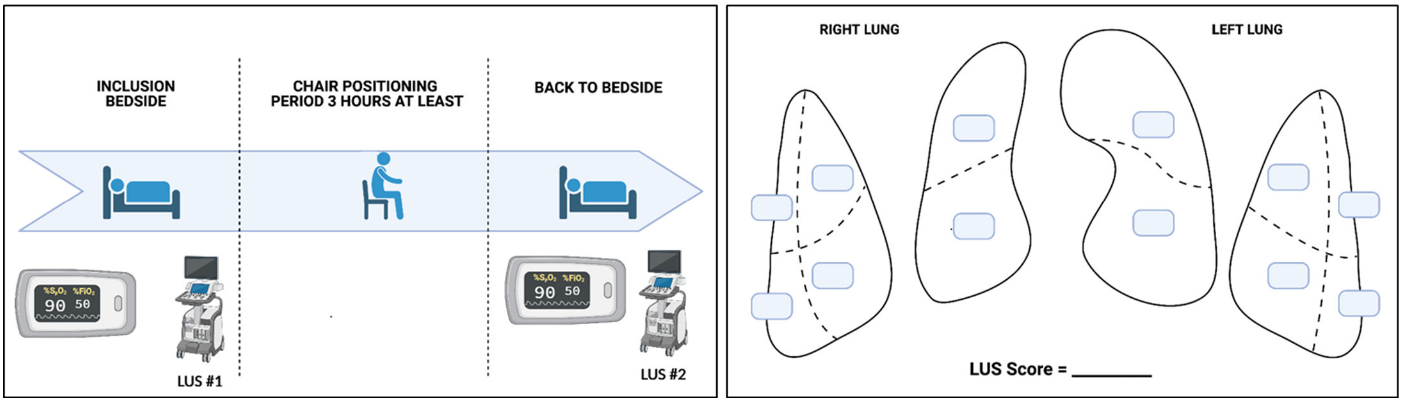

Figure S1. Study protocol, left pane : Lung ultrasound protocol. Right pane : Lung ultrasound examination – Areas (0: Normal or nearly normal (A-Pattern); 1: B-Pattern (B-lines > 3/field); 2: B-Pattern (crowed or coalescent); 3: Consolidation; E: Effusion).

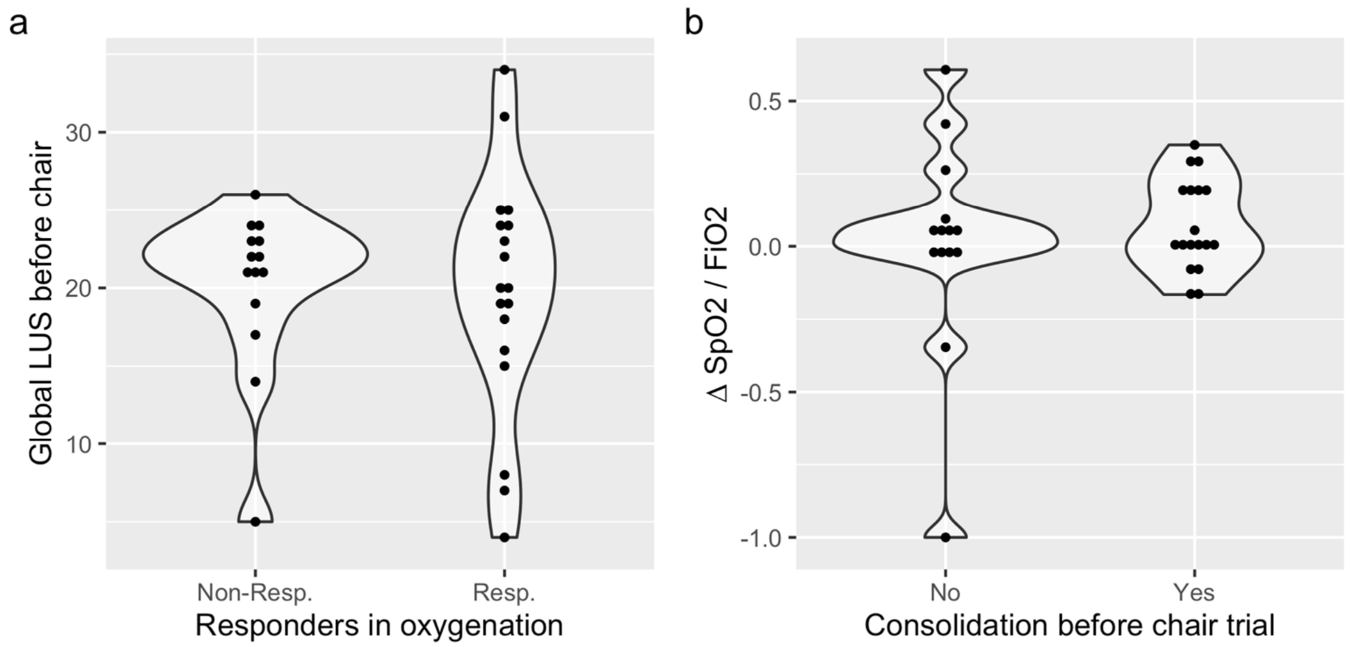

Figure S2. a / LUS scores before chair positioning session in oxygenation responders and non-responders; b/ Δ SpO_2:FiO_2 ratios in patients with and without ultrasonographic consolidation before chair trial session.

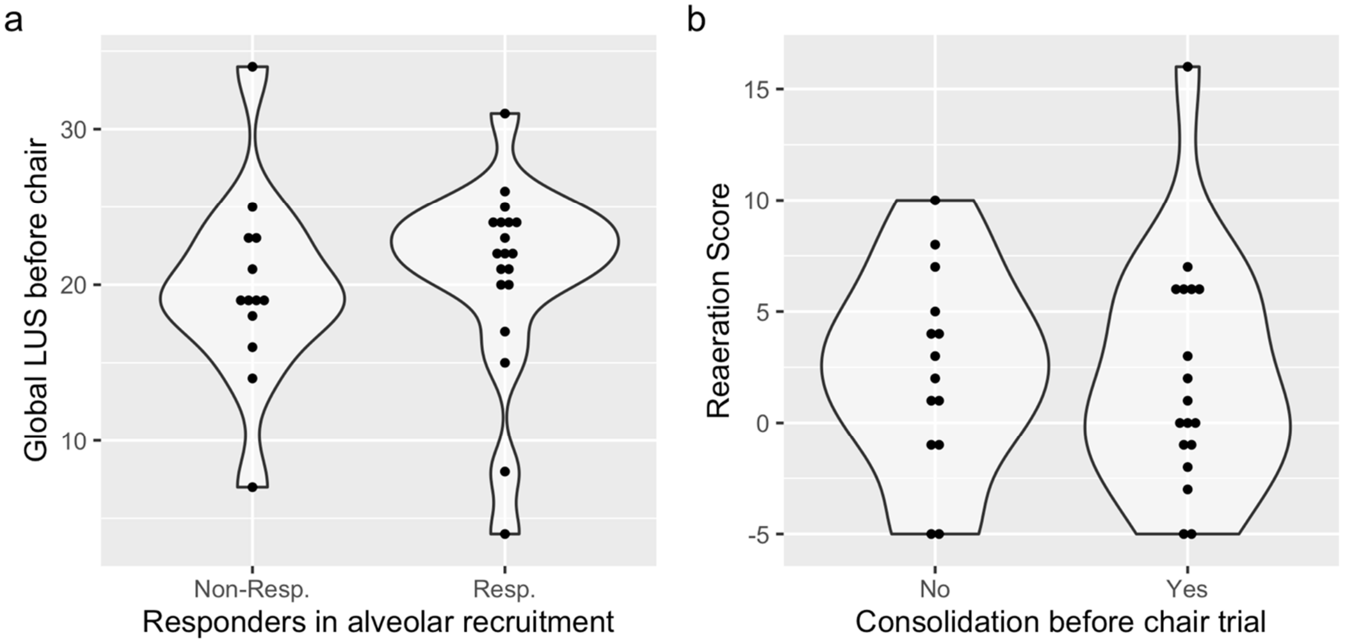

Figure S3. a / LUS scores before chair trial session in lung aeration responders and non-responders in terms; b/ Reaeration score in patients with and without ultrasonographic consolidations before chair trial.

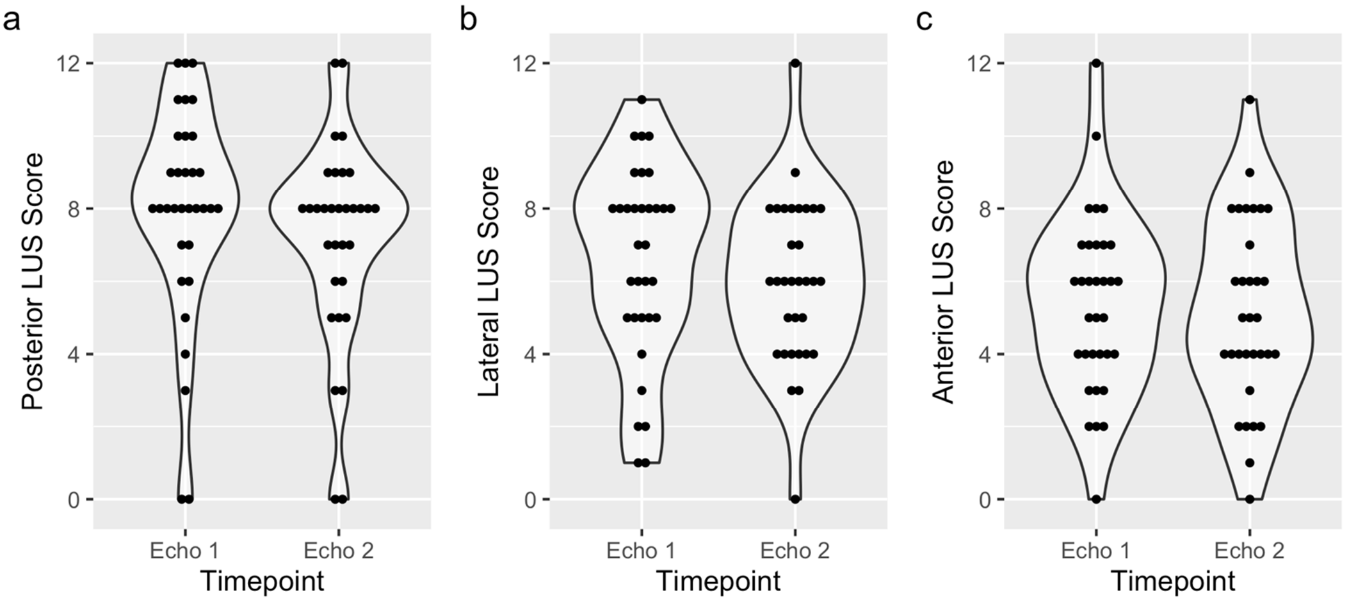

Figure S4. Regional LUS scores before and after chair positioning session: a/ Posterior LUS scores, b/ Lateral LUS scores, c/ Anterior LUS scores.

Table S1. Lung ultrasound reaeration score aimed at evaluating the effects of CP session on lung aeration.

Quantification of Reaeration			Quantification of Loss of Aeration		
1 point	3 points	5 points	- 5 points	- 3points	-1 point
B1 → N	B2 → N	C → N	N → C	N → B2	N → B1
B2 → B1	C → B1		0.4	B1 → C	B1 → B2
C → B2			0.5		B2 → C

B1, ultrasound lung cornets: B-Pattern > 3/fields; B2, B-pattern (crowed or coalescent); C, consolidation; N, normal pr nearly normal. First, ultrasound lung aeration (N, B1,B2, and C) was measured in each of the 12 regions of interest before the CP session (LUS1) and after the CP session (LUS2). Second, the score of lung aeration was calculated as the sum of each score characterizing each region according to the scale table.

Table S2. Outcomes according to oxygenation and lung aeration responders and non-responders groups.

A/ Oxygenation response

	Non-Responders on oxygenation (n=14)	Responders on oxygenation (n=18)	P-value
Invasive ventilation use during ICU stay, n (%)	5 (36)	3 (17)	0.4
Hospital Length of stay, Median [IQR], days	21 [12 - 23]	18 [10 - 23]	0.9
ICU Length of stay, Median [IQR], days	12 [9 - 14]	8 [6 - 14]	0.4
In-hospital mortality, n (%)	0 (0)	2 (11)	0.5
in-ICU mortality	0 (0)	2 (11)	0.6
28-day mortality, n (%)	0 (0)	2 (11)	0.6

B/ Lung aeration response

	Non-Responders on alveolar recruitment (n = 13)	Responders on alveolar recruitment (n = 19)	P-value
Invasive mechanical ventilation after inclusion, n (%)	5 (39)	2 (11)	0.1
Hospital length of stay, median [IQR], days	20 [16 - 24]	16 [10 - 22]	0.6
ICU length of stay, median [IQR], days	14 [10 - 15]	8 [6 - 10]	0.4
In-hospital mortality, n (%)	2 (15)	0 (0)	0.3
In-ICU mortality	2 (15)	0 (0)	0.3
28-day mortality, n (%)	2 (15)	0 (0)	0.3

Abbreviations: ICU, intensive care unit ; IQR, Interquartile range
